# Surgery of the Head

**Published:** 1918-10

**Authors:** 


**Surgery of the Head.** By CHARLES H. FRAZIER. *Progressive Medicine,* March 1, 1918.

*Gunshot Wounds of the Head.* All head injuries should be carried from the place where they fell to the place where they can be operated upon as rapidly as compatible with the military necessities of the moment.

All head injuries should be explored immediately upon arrival at the designated hospital, regardless of the hour of arrival, of the date and hour of the wound, of the statement on the diagnosis tag, of the clean appearance of the dressing, or of the patient's state of fatigue.

Head injuries should, whenever possible, be transported directly from the battlefield to the evacuation hospital, because :

*a*) Once operated upon they should not be subjected to further transportation until they are convalescent.

*b*) Because field ambulances and field hospitals are within range of artillery fire and the noise and concussion are very detrimental to such cases.

Systematic intervention in all penetrating wounds is necessary; only a very few cases make an exception to this rule.

The operation should be performed immediately.

It is advisable to examine the nervous system in every case, avoiding tiring the patient.

The radiological examination should in almost all cases precede the operation to obtain as exact a location as possible of the intra-cerebral projectile.

The osseous defect should be reduced to a minimum; it should be a few millimeters larger than the meningeal lesion.

All bony fragments projected into the brain should be extracted; the epidural space should also be carefully explored.

Deep-lying projectiles should be extracted early whenever their removal is possible without producing greater damage.

In cases of vast wounds with tearing of brain substance, drainage with gauze is extremely useful.

In all cases in which the wound is probably infected, the suture of the skin of the scalp should not be completely carried out.

The suture of the dura mater should be reduced to a minimum.

In lesions of the large sinuses, when hemorrhage cannot be stopped with simply tamponing, it is best to use a suture, limiting the use of the forcipressure to cases of extreme gravity and urgency.

Bandaging should be renewed as rarely as possible.

The rules of the most scrupulous asepsis should be followed in bandaging as well as in the operation.

The small cerebral hernias are reduced with compressive treatment; in large hernia all attempts at reduction should be omitted.

If the existence of a cerebral abscess has been diagnosed, it should at once be opened and drained.

The nursing of the patient should be most careful.

The patient should not be removed before a complete surgical cure.

The greater part of the deaths was due to the lesion itself; less frequently to meningo-encephalitis, suppurating cerebral hernia, and cerebral abscess.

*Technic for Removal of Bullets.* An unusual location of a bullet, which had lodged between the atlas and the base of the skull, gave Kanavel the opportunity to apply a novel technic for its removal through the mouth.

With the patient under anesthesia and the head thrown back, rubber tubes were introduced through the nostrils and the two ends brought out through the mouth, so that the soft palate could be held out of the way and the vault of the pharynx exposed. An incision about one-half inch in length was made to the right of the median line just behind and parallel to the posterior pillar on the right side and on a line with the atlas.

The muscular and connective tissues were separated by blunt dissection down to the bone and the tract of the bullet identified, but the bullet could not be seen. A silver wire was then introduced to the depth of the wound, with the aid of a fluoroscope, and the relation of the wire to the bullet was seen.

Resuming the operation it was found that the bullet had lodged in a pocket behind the upper part of the atlas, so that at times it lay between the atlas and the base of the skull and again in a pocket to the inner side of the atlas. Finally it was found at a depth of three and a half inches from the surface of the mucous membrane, and, after removal, the wound was painted with tincture of iodine and tamponed with gauze.

The technic of removing intrahemispherical foreign bodies differs entirely from that of removing a bullet within the hemispheres. In the first place, from the standpoint of localization, the cerebral falx serves as a very convenient and reliable guide, and, by following the falx, injury to the brain is avoided. The skull is opened with an osteoplastic flap, or with a trephine, 1 or 2 centimeters to one side of the median line to avoid the superior longitudinal sinus, either at the vertex, or 3 centimeters more posteriorly. A crucial incision is made in the dura and, with a flat retractor, the internal surface of the hemisphere is exposed. The missile is then searched for with the finger and removed with forceps.

